# Design, statistical analysis and sample size calculation of a phase IIb/III study of linagliptin versus voglibose and placebo

**DOI:** 10.1186/1745-6215-10-82

**Published:** 2009-09-05

**Authors:** Yoshiharu Horie, Naoyuki Hayashi, Klaus Dugi, Masahiro Takeuchi

**Affiliations:** 1 Biostatistics division, Graduate School of Kitasato University, 5-9-1 Shirokane, Minato-ku, Tokyo 108-8641, Japan; 2 Biostatistics Group, Nippon Boehringer Ingelheim Co., Ltd., 2-1-1 Osaki, Shinagawa-ku, Tokyo 141-6017, Japan; 3 Clinical Research Development group, Nippon Boehringer Ingelheim Co., Ltd., 2-1-1 Osaki, Shinagawa-ku, Tokyo 141-6017, Japan; 4 Medical Affairs Department, Boehringer Ingelheim Pharma GmbH & Co. KG, Mainz, Germany

## Abstract

**Background:**

Many patients with diabetes mellitus (DM) require a combination of antidiabetic drugs with complementary mechanisms of action to lower their hemoglobin A_1c _levels to achieve therapeutic targets and reduce the risk of cardiovascular complications. Linagliptin is a novel member of the dipeptidyl peptidase-4 (DPP-4) inhibitor class of antidiabetic drugs. DPP-4 inhibitors increase incretin (glucagon-like peptide-1 and gastric inhibitory polypeptide) levels, inhibit glucagon release and, more importantly, increase insulin secretion and inhibit gastric emptying. Currently, phase III clinical studies with linagliptin are underway to evaluate its clinical efficacy and safety. Linagliptin is expected to be one of the most appropriate therapies for Japanese patients with DM, as deficient insulin secretion is a greater concern than insulin resistance in this population. The number of patients with DM in Japan is increasing and this trend is predicted to continue. Several antidiabetic drugs are currently marketed in Japan; however there is no information describing the effective dose of linagliptin for Japanese patients with DM.

**Methods:**

This prospective, randomized, double-blind study will compare linagliptin with placebo over a 12-week period. The study has also been designed to evaluate the safety and efficacy of linagliptin by comparing it with another antidiabetic, voglibose, over a 26-week treatment period. Four treatment groups have been established for these comparisons. A phase IIb/III combined study design has been utilized for this purpose and the approach for calculating sample size is described.

**Discussion:**

This is the first phase IIb/III study to examine the long-term safety and efficacy of linagliptin in diabetes patients in the Japanese population.

**Trial registration:**

Clinicaltrials.gov (NCT00654381).

## Background

Diabetes mellitus affects around 171 million people worldwide and this figure is expected to double by the year 2030. Type 2 diabetes mellitus (T2DM) accounts for between 90 and 95% of all cases of diabetes [1]. In Japan, the Ministry of Health, Labour and Welfare estimates that there are approximately 8.2 million people "strongly suspected of having diabetes" and there are about 10.5 million people "for whom the possibility of diabetes cannot be ruled out", suggesting that there could be as many as 18.7 million Japanese sufferers. In other words, one in four adults in Japan may have or may be in the process of developing diabetes [2]. Estimates suggest that 2.3 million Japanese people are currently receiving medical care for diabetes and reports place diabetes as the third most common cause of outpatient visits for Japanese men. The disease is also associated with significant co-morbidity and mortality, and ranks as the tenth most likely cause of death for Japanese men and the ninth for Japanese women [2]. Type 2 diabetes mellitus is a progressive disease and, once diagnosed, the treatment pathway involves an increasingly complex combination of treatments as the disease worsens. Despite the various treatments available, there is no cure and treatment regimens often become more complex as patients demonstrate progressive deterioration of glycemic control. Therefore, there is a recognized clinical need for safe and effective new treatments for T2DM. Dipeptidyl peptidase-4 (DPP-4) inhibitors are one such class of antidiabetic agents that have recently been introduced into the clinical armamentarium.

The purpose of a drug development program is to establish through clinical trials whether a drug displays acceptable levels of safety and tolerability, and to establish whether the drug's actions are both beneficial and clinically relevant. In T2DM, the gold standard measure of efficacy is the impact of the antidiabetic agent on levels of glycosylated haemoglobin (HbA_1c_). Although no superior marker to HbA_1c _has yet been proposed, its use raises a number of methodological and ethical challenges in terms of conducting clinical trials. In general, antidiabetic agents effect changes in HbA_1c _levels by only a few percent (typically 0.5-2%). In contrast, patients vary widely both in their individual HbA_1c _levels (7-14%) and in disease response to various treatments. The inherent variability of HbA_1c _levels within the diabetic population and the relatively small changes caused by treatment dictate that clinical studies attempting to establish the efficacy of a new drug for T2DM need to involve relatively large numbers of subjects to demonstrate any statistically significant results confirming efficacy. Where studies compare the efficacy of one drug relative to another, the number of subjects required needs to be greater still. Pressure to recruit sufficient numbers of patients in a timely fashion makes it impractical to conduct such studies using treatment-naïve patients alone, where recruitment ideally should occur at, or around the time of diagnosis. The design of studies must therefore consider using patients already receiving treatment for diabetes. This introduces a further complicating factor, as previous exposure to an antidiabetic treatment may affect patient response to new treatment.

Although HbA_1c _serves as an excellent indicator of change in the general level of individual glycemic control, it takes 2 - 3 months for any treatment-related changes to be fully represented within the HbA_1c _measurement for any patient. This influences the design and implementation of clinical trials of T2DM in several ways. First, studies must be conducted either in treatment-naïve patients, or in patients who have undergone a suitable treatment washout period so that HbA_1c _values are allowed to return to pre-treatment levels. Withholding therapy known to benefit a patient for up to 2 - 3 months could be considered unethical. Second, in light of the turnover of red blood cells, patients need to be exposed to an investigational drug for up to 12 weeks to demonstrate the full effect of the drug on HbA_1c _levels. Under these circumstances, if a study employs a standard placebo-controlled design, patients in the placebo group might go for up to 24 weeks without receiving diabetes treatment. Thus, the challenge in conducting clinical trials in T2DM is to design suitably powered efficacy studies that are both practicable and ethical. It is proving increasingly difficult to deliver such studies using traditional randomized, placebo-controlled designs. The employment of more complex trial designs that are appropriately powered may serve to address some of these challenges.

Dipeptidyl peptidase-4 (DPP-4) inhibitors are a new and promising class of agents for the treatment of T2DM. Linagliptin is a novel member of the DPP-4 inhibitor class that may offer added clinical benefits over similar agents. These include a long half-life consistent with once-daily dosing, non-renal excretion that will not require dose adjustment following loss of renal function and excellent safety and tolerability profiles [3-6]. As DPP-4 inhibitors increase insulin secretion, they are expected to become an important treatment for T2DM in Japanese patients, who tend to be leaner than patients in Western countries. Lean patients are more likely to be older at diagnosis, possess an immune component to their T2DM, and demonstrate certain pathophysiological characteristics, notably less insulin resistance and poorer insulin secretory capacity [7]. The present study proposes to investigate the effect of linagliptin on HbA_1c _levels in a Japanese population at two different doses (5 mg and 10 mg). It is possible that these patients will respond differently than Caucasian patients due to genetic differences. Differences in the pharmacokinetic and/or pharmacodynamic behaviour of drugs between Japanese subjects and subjects from other ethnic backgrounds have been observed, for example for the oral contraceptive Org 30659 [8] and the anti-cholesterol agent, rosuvastatin [9]. As a consequence, guidance from the Pharmaceutical and Medical Devices Agency (PMDA) in Japan recommends that the dose-response relationship for any new drug is confirmed in the Japanese population and that treatment is evaluated in 'adequate numbers of Japanese cases' [10].

The primary objectives of the proposed study are to compare the efficacy of linagliptin versus placebo in lowering HbA_1c _in a 12-week study conducted in Japanese patients with T2DM, and to examine the efficacy of linagliptin versus the antidiabetic agent voglibose. The study will also evaluate the long-term safety profile of linagliptin for up to 52 weeks. The study design, primary endpoints, patient inclusion/exclusion criteria and statistical methods used to calculate the study sample size are described.

## Methods/Design

### Study Setting

This is a randomized, double-blind study with both a placebo- and an active-controlled parallel group. It is conducted in 47 centers in Japan. To be eligible for study enrollment, male or female subjects are required to be Japanese patients aged 20-80 years with T2DM (baseline HbA_1c _levels of 7.0-10.0%). Patients taking oral anti-hyperglycemic drugs at screening are eligible to participate if they have a mean HbA_1c _value of > 7 - < 9% during the washout period. Other inclusion criteria include a stable HbA_1c _value (less than 10% variation during the last 2 weeks of the 4-week washout period) and a body mass index less than 40 kg/m^2 ^at randomization. Patients are excluded from the study if they violate these inclusion criteria or if they meet the exclusion criteria specified in the protocol.

All patients must give written consent to participate in the study, which has been approved by an Institutional Review Board at each center. This study is registered with the US National Institutes of Health website: ClinicalTrials.gov (NCT00654381).

### Study Stages

**Figure 1:**
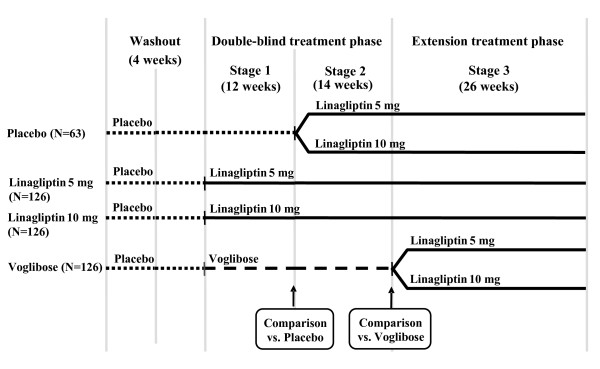
**Study Design**.

This study includes three different stages, each with specific objectives, and will utilize four treatment arms (A-D) (see Figure 1). The objective of Stage 1 is to compare changes in HbA_1c _levels in patients receiving linagliptin with those receiving placebo at 12 weeks. The objective of Stage 2 is to compare changes in HbA_1c _levels in patients receiving linagliptin with those receiving voglibose after 26 weeks of treatment. The objective of Stage 3 is to determine the long-term safety and tolerability of linagliptin.

Initially, patients will undergo a washout period where they receive placebo for 4 weeks after providing informed consent. Following the washout period, eligible patients will be randomized to one of four treatment arms (placebo [A]; linagliptin 5 mg [B]; linagliptin 10 mg [C]; or voglibose 0.6 mg [D]). Patients randomized to Group A will receive a placebo for 12 weeks after which they will receive linagliptin for 40 weeks; patients in the Group B (linagliptin 5 mg) and Group C (linagliptin 10 mg) treatment arms will receive the study drug for up to 52 weeks. Patients randomized to Group D (voglibose 0.6 mg) will receive the treatment for 26 weeks after which they will be switched to linagliptin for 26 weeks (Figure 1). The treatment change to linagliptin in Groups A and D involves an additional patient randomization to either 5 mg or 10 mg, from which data on the long-term safety and tolerability of linagliptin will be collected. The doses of linagliptin used in this study are based on previous reports of DPP-4 activity in Japanese T2DM patients [11].

### Study Endpoints

The proposed comparison of efficacy between linagliptin (5 and 10 mg) and both placebo and voglibose is based on changes in HbA_1c _levels from baseline at 12 and 26 weeks, respectively. The primary comparison between linagliptin 10 mg and placebo is planned at 12 weeks, and the comparison between linagliptin 10 mg and voglibose 0.6 mg is planned at 26 weeks. To investigate dose-response relationships, a subsequent comparison is made at those time points between linagliptin 5 mg and placebo (12 weeks) and voglibose 0.6 mg (26 weeks).

Other parameters of interest measured will include inhibition of plasma DPP-4 activity, the proinsulin/insulin ratio, the homeostasis model assessment (HOMA) indices for insulin resistance and insulin secretion, body weight, waist circumference and plasma concentrations of linagliptin. Adverse event, safety, laboratory and hematology data will be collected during the study and will form part of the secondary endpoint data for determining the long-term safety profile of linagliptin.

Patient reported outcomes will be assessed using the Diabetes Treatment Satisfaction Questionnaire (DTSQ) modified by Ishii [12], consisting of 6 items assessing treatment satisfaction and 2 items assessing the perceived frequencies of hyperglycaemia and hypoglycaemia.

### Study Randomization

Four treatment arms are used to investigate the effective dose and safety profile of linagliptin in the study population. Randomization will be based on the Zelen rule [13] and follow a predefined allocation ratio of 2:2:2:1 (linagliptin 5 mg: linagliptin 10 mg: voglibose: placebo). Allocation to treatment groups is balanced for the potentially confounding effects of patient background and baseline characteristics, HbA_1c _(stratified into two categories; < 8.5% and ≥ 8.5%), the number of pre-study use of antidiabetic drugs, gender and study site.

Treatment allocation is conducted by a sponsor-independent contractor. Following informed consent, the investigator completes a registration form that is sent to the allocation center. The registration form includes data on the subjects' inclusion and exclusion criteria. After receiving the registration form, the allocation center sends a registration report to the investigator. The investigator confirms that the patient satisfies the study criteria and sends a second registration form to the allocation center once the 4-week washout period is completed. The allocation center randomizes the patient according to the pre-defined criteria above and this information is provided to the investigator. Randomization is done once at the enrollment of study. At the randomization after the enrollment, patients are assigned, to 6 groups: placebo - linagliptin 5 mg, placebo - linagliptin 10 mg, linagliptin 5 mg, linagliptin 10 mg, voglibose - linagliptin 5 mg, and voglibose - linagliptin 10 mg. Therefore all patients ultimately take either linagliptin 5 mg or 10 mg, unless they withdraw or drop out the study.

### Statistical analysis

The statistical analyses are performed on an intention-to-treat basis. The full analysis dataset (FAS) is described in the ICH E9 guidelines [14], and is defined as the proposed analysis dataset for efficacy. The procedures followed are described below.

#### Analysis for the primary endpoint

Baseline glycosylated hemoglobin levels and the number of antidiabetic drugs used before study enrollment are included in the model as covariates.

Changes in HbA_1c _level from baseline to 12 weeks of linagliptin treatment are analyzed in accordance with the model below, in order to compare with placebo in the double-blind treatment phase:



Where:

*y*_*ijk *_: change in HbA_1c _at 12 weeks from baseline

*BHbA1c*_*i *_: HbA_1c _of i-th patient at baseline (i = 1, 2,..., n)

*Dose*_*j *_: j-th dose group (j = 0: placebo, 1: 5 mg, 2: 10 mg)

*Diabtreat*_*k *_: number of previously used antidiabetic drugs (k = 0: no treatment, 1: one antidiabetic drug, 2: two antidiabetic drugs)

Comparisons conducted at 12 weeks are between linagliptin and placebo. Patients who received placebo for 12 weeks and are then randomized to receive either linagliptin 5 mg or 10 mg are excluded from this analysis. Values of HbA_1c _following treatment with linagliptin are to be compared with those for voglibose after 26 weeks if linagliptin treatment fails to achieve a statistically significant difference in HbA_1c _levels relative to placebo (primary comparison dose at 12 weeks). The dosing schedule for linagliptin increases from 5 mg to 10 mg during study if linagliptin 5 mg fails to demonstrate superiority to placebo at 12 weeks. In addition, the study stops if neither of the linagliptin arms shows statistically significant differences from placebo in HbA_1c _levels at 12 weeks.

The model for HbA_1c _at 26 weeks is:



Where:

*y*_*ijk *_: Change in HbA_1c _at 26 weeks from baseline

*HbA1c*_*i *_: HbA_1c _of i-th patient at baseline (i = 1, 2,..., m)

*Drug*_*j*_: j-th treatment group (j = 1:5 mg, 2:10 mg, 3:voglibose)

*Diabtreat*_*k *_: number of previously used antidiabetic drugs (k = 0:no treatment, 1:one antidiabetic drug, 2:two antidiabetic drugs)

Comparisons for HbA_1c _between groups are performed weekly by the closed testing procedure [15]. No formal analysis will be performed for safety and tolerability data collected during the three stages of the study. Data will be summarized in terms of treatment received.

### Sample size

Sample size calculations are performed using nQuery Advisor^® ^6.0 (Statistical Solutions Ltd, Ireland) software on a Windows-based personal computer. On the basis of clinical studies conducted in Japan and other countries, it is estimated that the overall mean change in HbA_1c _levels after treatment with linagliptin 5 mg QD for 12 weeks will be approximately 0.5% compared with 0% in placebo. This will provide an overall mean between-groups treatment difference of 0.5%. Experience also suggests that the standard deviation of this difference will be approximately 0.9%. Employing an accepted one-sided statistical significance threshold of 2.5% and taking into account the weighting for placebo and linagliptin group randomization ratios (1:2), it is possible to predict a 90% power for the study with sample sizes of no less than 52 patients in the placebo group and no less than 104 patients in the linagliptin groups.

Assuming that HbA_1c _levels for the linagliptin treatment groups will continue to fall with ongoing therapy, estimates suggest that the mean change in HbA_1c _levels from baseline after a 26 week treatment with linagliptin 5 mg, will be approximately 0.7%, compared with 0.25% for voglibose 0.6 mg. It is expected that this difference between treatments will be reflected in a mean difference in HbA_1c _levels from baseline of 0.45% [16]. With a standard deviation of the difference at 1.0% and the level of significance set at 2.5% (one-sided analysis), sample sizes of 105 or more in each group provide a power greater than 90%.

Experience derived from previous clinical trials in patients with T2DM suggests that a discontinuation/dropout rate of approximately 15% can be expected during the current 52-week trial. Assuming that the present study will experience a similar level of subject discontinuation, it is possible to adjust the recruitment targets to maintain study power in anticipation of similar attrition levels. A target sample size of 63 patients in the placebo group and 126 patients each in the linagliptin and voglibose treatment groups (a total of 441 patients overall) is therefore required to power this study appropriately to achieve its primary objectives.

The sample size in the linagliptin group also appears to satisfy the requirements for evaluation of safety in the clinical development of new drugs expected for long-term use in non-fatal diseases [17].

## Discussion

The design of the present study is adopted as a means of addressing some of the ethical and methodological challenges in studying new drugs in T2DM populations.

Glycosylated hemoglobin has emerged as the accepted marker of glycemic control and clinical efficacy in studies of diabetes [18]. The nature of HbA_1c_, particularly its 2 - 3 month period for turnover, tends to dictate the time course of T2DM clinical trials. Similarly the inter-patient variability in terms of the levels of HbA_1c _and its response to various treatments dictate that studies must recruit relatively large numbers of patients to demonstrate statistically significant changes in HbA_1c _compared with placebo [19]. The challenge in recruiting sufficient numbers of patients is still greater if a study intends to compare the efficacy of two or more different treatments. To make treatment studies in T2DM more practical, it is necessary not to limit the patient population only to treatment-naïve patients. This introduces the potentially confounding factor of prior exposure to antidiabetic drugs and its potential impact on both baseline values and treatment response. To minimize this effect it is necessary to provide a sufficient treatment washout period to reduce the impact of the previous treatment. However, to allow HbA_1c _to return to baseline levels it would be necessary for the washout period to last 2-3 months, and if the study design involves a placebo arm, this would mean a significant number of patients going without treatment for an extended period.

Withholding treatment from patients for a prolonged period might be considered unethical. Therefore, the present study uses a 4-week washout period. Clearly, 4 weeks is insufficient for HbA_1c _levels to return to the baseline level before initiation of the previous treatment. However, by adopting this approach it is hoped that we may address some of the concerns frequently raised about prolonged treatment withdrawal in clinical trials [20]. The statistical analysis plan and analysis of the power of the study, therefore, take into account the fact that HbA_1c _levels at treatment initiation will most likely incorporate an aspect of the pre-study treatment response, the antidiabetic drug used and the previous extent of disease control per patient.

The study design was selected to reduce the number of patients required to undergo a prolonged placebo exposure by employing an imbalanced randomization (placebo:linagliptin 5 mg:linagliptin 10 mg:voglibose = 1:2:2:2). Using this biased treatment allocation approach, it is possible to minimize the potentially unethical exposure of patients to placebo while maintaining sufficient power to complete the primary statistical comparison. Data after 16 weeks of placebo treatment can be useful for determining a true baseline (treatment naïvety) because any underlying glycemic controlling effects of prior antidiabetic treatment should have had sufficient time to reverse. The 16 week data for glycosylated HbA_1c _levels in the placebo group will not be included in any comparison, but instead will be viewed as an indication of the study population's baseline HbA_1c _values in the absence of the potentially confounding residual effects of previous therapies. A similar number of patients are needed to demonstrate the non-inferiority or superiority of linagliptin to placebo under imbalanced randomization and to voglibose. The study design can also be sufficiently powered for detecting statistically significant differences between linagliptin and placebo or voglibose. Using the methods described, we have calculated that this can be achieved without markedly increasing the number of subjects needed.

The efficacy of short term linagliptin treatment in Japanese T2DM patients was reported by Kanada et al [11]. We set the one-sided hypotheses (2.5% statistically significant level) in the present study as the efficacy of linagliptin is expected to show significant superiority to placebo or voglibose. The efficacy of linagliptin is established only in the case that linagliptin shows superiority to placebo and voglibose simultaneously. Therefore, adjustment for multiple hypotheses is not carried out.

Our study design may also facilitate recruitment relative to other methods. In clinical studies of antidiabetic drugs as monotherapy, all patients randomized to placebo groups receive only placebo; therefore, there are no perceived benefits for these patients. The use of placebo-only groups in clinical studies may cause patients to hesitate when asked to participate, as they are aware that they have a chance of receiving inadequate treatment during the trial. In the present study, patients receive placebo for a maximum of 16 weeks after which they are given active treatment. This design may serve to aid patient recruitment in clinical studies. Similarly, study designs which incorporate multiple objectives within a single study may reduce the burden of recruitment on the development program by maximizing the information derived from every study. This study investigates the efficacy of two different doses of linagliptin versus placebo (at 12 weeks), compares the relative efficacy of linagliptin versus voglibose (at 26 weeks) and provides long-term (52 week) safety data. Combining the objectives into a single study effectively cuts the pre-study clinical workup that would be needed if these investigations were conducted as three separate studies, thus reducing the patient recruitment burden. Other ongoing Phase III trials are evaluating linagliptin as both monotherapy and as add-on therapy to commonly used diabetes medications. It is anticipated that these trials combined with the results of the present study will support the suitability of linagliptin as a further treatment option for type 2 diabetes patients in Japan.

In conclusion, a continuous flow study design has been applied to investigate three distinct areas of interest related to the novel DPP-4 inhibitor linagliptin in Japanese diabetes patients. The design described offers several logistical and ethical advantages, while remaining adequately powered to generate informative results for this patient population. Full results from this trial will be published in 2010.

## Competing interests

This trial was supported by Boehringer Ingelheim. With the exception of MT, all of the authors are full-time employees of Boehringer Ingelheim. Involvement in this trial does not conflict with the professional activities of MT.

## Authors' contributions

All authors read and approved the final manuscript. YH as an investigator in the study, drafted the manuscript and has made a substantial contribution to acquisition of data.
